# Pregnancy outcomes after living kidney donation from a nationwide population-based cohort study from Korea

**DOI:** 10.1038/s41598-022-27094-x

**Published:** 2022-12-27

**Authors:** Juhan Lee, Kyu Ha Huh, So Ra Yoon, Soo Yeun Lee, Hyung Soon Lee

**Affiliations:** 1 Department of Surgery, Yonsei University College of Medicine, Seoul, Republic of Korea; 2 Research Institute, National Health Insurance Service Ilsan Hospital, Goyang, Republic of Korea; 3 Department of Surgery, National Health Insurance Service Ilsan Hospital, 100 Ilsan-Ro, Ilsandong-Gu, Goyang, 10444 Republic of Korea

**Keywords:** Kidney, Kidney diseases

## Abstract

While most living kidney donors experience good outcomes and high rates of satisfaction, kidney donation can increase the risk of gestational hypertension or preeclampsia. However, pregnancy outcomes in non-white donors are limited. We conducted a nationwide cohort study of 112 living kidney donors and 672 matched healthy non-donors using the Korean National Health Insurance Claims Database. Donors and healthy non-donors were matched according to age, year of cohort entry, residency, income, number of pregnancies, and the time to the first pregnancy after cohort entry. We assessed pregnancy outcomes of live kidney donors compared with matched healthy non-donors using the nationwide database. Gestational hypertension or preeclampsia was more common in kidney donors than in non-donors (8.9% vs. 1.8%; adjusted odds ratio, 2.68; 95% confidence interval, 1.11–6.50). However, the incidence of severe gestational hypertension or preeclampsia that required antihypertensive medication was comparable (2.7% vs. 0.9%; *P* = 0.121). The time from donation to delivery within 5 years and primiparity were risk factors for preeclampsia in donors. Low birth weight, stillbirth, and ectopic pregnancy were not significantly different between the two groups. Maternal death occurred in two non-donor cases, but none occurred in donors compared to non-donors. Our findings indicate that kidney donors are associated with an increased risk of gestational hypertension or preeclampsia than matched healthy non-donors. However, the probabilities of serious maternal and fetal outcomes remained low and are not increased significantly after kidney donation.

## Introduction

Living donor kidney transplant (KT) is the most effective treatment for eligible patients with end-stage kidney disease (ESKD), offering superior outcomes compared to deceased donor transplant^1,2^. There have been increasing efforts to better define the risks of ESKD after kidney donation^3–6^. Recent studies comparing to individuals selected for baseline good health suggest that kidney donation is associated with an increase in the risk of ESKD, although the absolute post-donation risk remains very low^7,8^.

In addition to ESKD risk, female donor candidates have concerns regarding the safety of future pregnancies after donation^9^. Prior studies have reported higher rates of hypertensive pregnancy disorders in women with preexisting kidney disease^10–12^. In kidney donors, marginal increase in blood pressure or acute kidney injury as well as reduced overall kidney function might increase the risk of pregnancy-related complications. Several studies reported an increased risk of gestational hypertension or preeclampsia after donation^13,14^. Based on these results, it is recommended that potential donors with reproductive potential should be counseled about the risk of gestational hypertension or preeclampsia^15,16^.

However, previous studies have been conducted in a limited number of patients and most women were white^17^. Another two previous studies have compared gestational hypertension or preeclampsia that occur during pregnancy before and after donation^14,18^. Therefore, there are limitations in the available information on pregnancy outcomes after kidney donation in non-white donors^19^.

With a high proportion of female donor and increasing need for living donor KT worldwide, there is an urgent need to evaluate the pregnancy outcomes in kidney donors. The present study aimed to investigate the pregnancy outcomes of live kidney donors compared with matched healthy non-donors using the nationwide database. We also explored risk factors for gestational hypertension or preeclampsia after kidney donation.

## Methods

### Data sources and study participants

This nationwide study was based on the Korean National Health Insurance (NHI) claims database. The Korean NHI system is a nationwide insurance system operated by the Korean government and covers up to 98% of the Korean population. The Korean NHI claims database contains patients’ sociodemographic information, their use of inpatient and outpatient services, diagnosis, and medical treatment. The International Classification of Diseases 10th Revision (ICD-10) code was used to record diagnoses in this database. It has been shown that the database provides reliable estimates of the prevalence of certain diseases in Korea^20–22^.

This study included women aged > 19 years old who had at least one delivery between 2007 and 2018 (ICD-10: R4351, R4335, R4356, R4358, R3131, R3138, R3141, R3143, R3146, R3148, R4361, R4362, R4370, R4376, R4376, R4376, R4379, R7380, R4514, R4516, R4507, R4508, R4509. R4510, R4514, R4516, R4517, R4518, R1519, R4520, R5001, R5002). Among them, we excluded women who had diagnostic codes for hypertension or diabetes mellitus during the previous 5 years. We identified donors who donated a kidney between 2007 and 2016 by searching the standardized code for donor nephrectomy (R3272) and healthy kidney donor (Z52.4). We excluded donors with multi-fetal gestation (more than one fetus at a time) or a history of organ transplant (ICD-10: O30, O31, O632, O661, O663 and O692 for multi-fetal gestation; R3280, Q8040-Q8050, Q8140-Q8150, Q8061, Q8062, Q8080, Q8101, Q8102, Q8103, Q8121, Q8122, Q8123 for history of organ transplant). Each donor’s nephrectomy date served as her cohort-entry date.

The control group was selected among pregnant women who had no history of hypertension, diabetes mellitus, or organ donation during the same period. We also excluded women who had multi-fetal gestation or a history of organ transplant. We randomly assigned a cohort-entry date to all women according to the distribution of cohort-entry dates of donors (January 1, 2007, to December 31, 2017). We then matched six controls per donor on the basis of age at entry, year of cohort entry, residency, income, number of pregnancies, and the time to the first pregnancy after cohort entry.

This study was approved by the Institutional Review Board of National Health Insurance Service Ilsan Hospital (2021-04-009). The need for written informed consent was waived by the board owing to the retrospective nature of the study. All methods were performed in accordance with the relevant guidelines and regulations.

### Definition and study endpoints

The primary study endpoint was gestational hypertension or preeclampsia. Secondary endpoints were severe gestational hypertension or preeclampsia, parity, low birth weight (< 2500 g), stillbirth, ectopic pregnancy, and maternal death. Women were followed until death, immigration from Republic of Korea, or the end of the study period (December 31, 2019).

We investigated pregnancy outcomes using ICD-10 code and prescription data from the Korean NHI claims database (ICD-10: O10, O13, and O16 for gestational hypertension; O11 and O14 for preeclampsia; O15 for eclampsia; O365 for low birth weight (< 2500 g); O364 for stillbirth; O00 for ectopic pregnancy; O03, O04, O05, O06, O07 for maternal death). Gestational hypertension was defined as new onset hypertension (blood pressure ≥ 140/90) after 20 weeks of gestation in the absence of proteinuria. Preeclampsia was defined as gestational hypertension with proteinuria (≥ 0.3 g per day or ≥  + 1 on a standard urine test strip). The operational definition of severe gestational hypertension or preeclampsia is defined as a diagnostic code with a history of prescription of antihypertensive medications. If there were one or more prescriptions during the target period (from 10 months before the delivery day to the delivery day), it was considered that the medication was used.

### Confounding variables

We investigated age at entry, time from entry to delivery, number of deliveries, residence, and income. The income quintiles of each insured person represent the economic status of the persons and are based on the amount of health insurance payments. Comorbid conditions of the patients were evaluated on the basis of claim codes within 5 years before the cohort entry using the Charlson comorbidity index^23^.

### Statistical analysis

To compare pregnancy outcomes between living kidney donors and non-donors, we performed propensity score matching to adjust for differences in patient characteristics. Propensity scores were estimated using multiple logistic regression analysis. Regression analysis predicted the probability that each patient would be treated based on age at entry, total number of pregnancies, residency, and income. The discrimination and calibration abilities of the propensity score models were assessed with the C-statistic and Hosmer–Lemeshow statistic. After the propensity score-matched sample was formed, we assessed the baseline variable balance between the two propensity-matched cohort groups.

Continuous variables were compared using the paired t-test or the Wilcoxon signed rank test, as appropriate, and categorical variables were compared with the McNemar’s or marginal homogeneity test, as appropriate^24^. The Mann–Whitney U and Wilcoxon signed rank tests were used to compare continuous variables between subject groups, and the chi-square test was used to compare categorical variables. Cox proportional hazards analysis was used to compare pregnancy outcomes and risk factors for gestational hypertension or preeclampsia between donors and non-donors. We adjusted the comorbid conditions of the patients using the Charlson comorbidity index. All statistical analyses were performed with SAS 9.2 (SAS Institute Inc., Cary, NC, USA) and RStudio v1.1.463 (RStudio Inc., Boston, MA, USA), and *P* values of less than 0.05 were considered statistically significant.

## Results

### Study population

A total of 4,248,899 women with a history of pregnancy and without diagnostic codes for hypertension and diabetes mellitus between 2007 and 2018 were screened for the study. Among them, 647 kidney donors were identified using healthy kidney donor diagnostic code and donor nephrectomy procedure code. Five living donors with a history of multi-fetal gestation and one donor with solid organ transplant after kidney donation were excluded. Among the remaining 641 donors, 112 with a history of delivery after kidney donation were selected.

**Figure 1:**
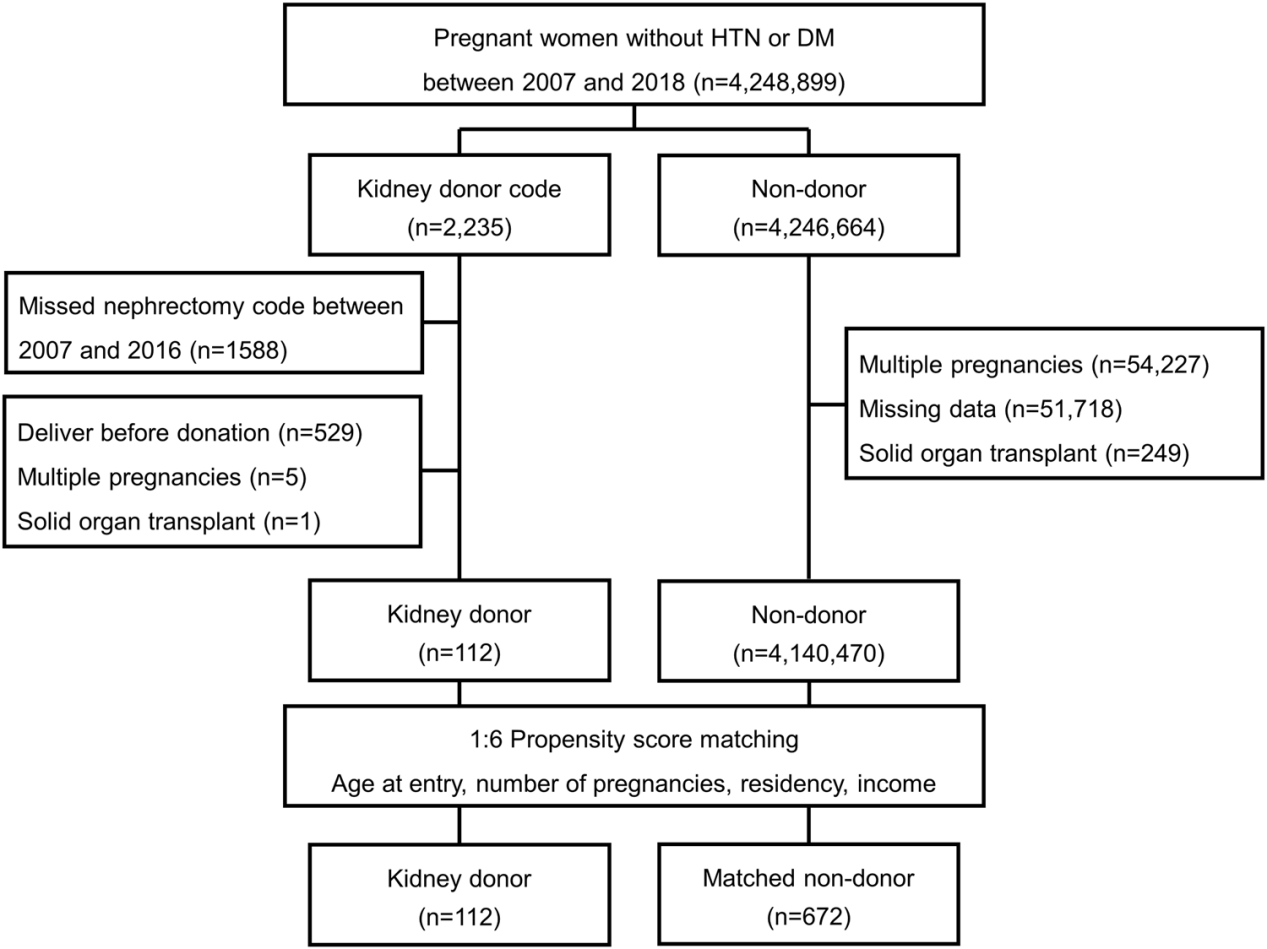
Patient selection schematic.

Of the 4,246,664 pregnant women without history of kidney donation, 54,227 with a history of multi-fetal gestation, 249 with a history of solid organ transplant (liver, 199; pancreas, 4; heart, 23; lung, 22; small bowel, 1), and 51,718 with missing data were excluded. A total of 672 were selected for the control group based on age at time of entry, total number of pregnancies, residency, and income (Fig. 1).

### Baseline characteristics

During the study period, 150 pregnancies after kidney donation from 112 donors were identified from the Korean NHI claims database. Kidney donors were younger and more likely to live in urban areas than in overall non-donors. Mean time from donation to delivery was 4.1 ± 2.2 years. After propensity score matching, 672 non-donors were included. Baseline characteristics of both overall and matched non-donors compared to donors are shown in Table 1. Propensity score matching was adequate and all covariates were well balanced between the two groups.

**Table 1:** Baseline characteristics of donors and non-donors before and after propensity score matching.

	Before matching			After matching		
	Donors (n = 112)	Non-donors (n = 4,140,470)	*p*-value	Donors (n = 112)	Non-donors (n = 672)	*p*-value
Age at entry (y)	26.07 ± 3.62	30.47 ± 4.13	< 0.001	26.07 ± 3.62	26.07 ± 3.64	> 0.999
Residence			< 0.001			> 0.999
Seoul	20 (17.9%)	930,267 (22.5%)		20 (17.9%)	120 (17.9%)	
Metropolitan city	42 (37.5%)	1,038,670 (25.1%)		42 (37.5%)	252 (37.5%)	
Province	45 (40.2%)	1,904,462 (46.0%)		45 (40.2%)	270 (40.2%)	
Others	5 (4.4%)	267,071 (6.4%)		5 (4.4%)	30 (4.4%)	
Income quintile			< 0.001			> 0.999
First (lowest)	6 (5.36%)	19,243 (0.5%)		6 (5.36%)	36 (5.36%)	
Second	21 (18.75%)	750,925 (18.1%)		21 (18.75%)	126 (18.75%)	
Third	36 (32.14%)	1,181,423 (28.5%)		36 (32.14%)	216 (32.14%)	
Fourth	31 (27.68%)	1,457,450 (35.2%)		31 (27.68%)	186 (27.68%)	
Fifth (highest)	18 (16.07%)	731,429 (17.7%)		18 (16.07%)	108 (16.07%)	
Total number of deliveries			< 0.001			> 0.999
1 (primipara)	66 (58.93%)	2,195,834 (53.0%)		66 (58.93%)	396 (58.93%)	
2 (multipara)	38 (33.93%)	1,653,948 (39.7%)		38 (33.93%)	228 (33.93%)	
3 (multipara)	8 (7.14%)	264,937 (6.4%)		8 (7.14%)	48 (7.14%)	
≥ 4 (multipara)		25,751 (0.6%)				

### Pregnancy outcomes between living kidney donors and matched non-donors

The pregnancy outcomes between donors and matched non-donors are shown in Table 2. The rate of gestational hypertension or preeclampsia was 1.79% in non-donors, but 8.93% in donors, which was significantly higher in living kidney donors (adjusted odds ratio, 2.68; 95% confidence interval, 1.11–6.50; *P* = 0.029). However, there was no significant difference in severe gestational hypertension or preeclampsia that required antihypertensive medication between the two groups (0.9% vs. 2.7%; *P* = 0.121).

**Table 2:** Pregnancy outcomes between donors and matched non-donors.

Outcomes	Donors (n = 112)	Non-donors (n = 672)	aOR (95% CI)	*p*-value
Gestational hypertension or preeclampsia	10 (8.9%)	12 (1.8%)	2.68 (1.11–6.50)	0.029
Severe gestational hypertension or preeclampsia	3 (2.7%)	6 (0.9%)	2.99 (0.75–11.96)	0.121
Low birth weight	2 (1.8%)	11 (1.6%)	3.35 (0.82–13.68)	0.093
Stillbirth	1 (0.9%)	5 (0.7%)	0.45 (0.05–4.31)	0.488
Ectopic pregnancy	9 (8.0%)	37 (4.7%)	1.02 (0.87–1.21)	0.462
Maternal death	0	2 (0.3%)	–	–
Blood transfusion	7 (6.3%)	11 (1.6%)	38.24 (13.35–109.54)	< 0.001

*aOR* adjusted odds ratio, *95% CI* 95% confidence interval.

The incidence rates of low birth weight, stillbirth, and ectopic pregnancy were not significantly different between the two groups. Maternal death occurred in two cases of non-donors, but none occurred in donors.

### Risk factors for gestational hypertension and preeclampsia in kidney donors

The risk factors for gestational hypertension or preeclampsia are shown in Table 3. The time from donation to delivery within 5 years and primiparity were significantly associated with the increased risk of preeclampsia.

**Table 3:** Risk factors for gestation hypertension or preeclampsia in living kidney donors.

	Donors (n = 112)	Non-donors (n = 672)	aOR (95% CI)	*p*-value
**Gestational hypertension**				
Age at first delivery after donation				
≤ 32 years	2/65(3.1%)	4/390 (1.0%)	1.79 (0.27–12.02)	0.55
> 32 years	0/47	5/282 (2.1%)	–	–
Time from entry to delivery				
≤ 5 years	2/66 (3.0%)	5/410 (1.2%)	1.38 (0.20–9.55)	0.746
> 5 years	0/46	4/262 (1.9%)	–	–
Number of deliveries				
1 (primipara)	1/66 (1.5%)	6/396 (1.8%)	–	–
2 (multipara)	1/38 (2.6%)	3/228 (1.3%)	1.43 (0.13–16.23)	0.773
3 (multipara)	0/8	0/48	–	–
**Preeclampsia**				
Age at first delivery after donation				
≤ 32 years	3/65 (4.6%)	0/390	–	–
> 32 years	5/47 (10.6%)	3/282 (1.1%)	4.72 (0.96–23.28)	0.057
Time from entry to delivery				
≤ 5 years	6/66 (9.1%)	2/410 (0.5%)	7.86 (1.25–49.22)	0.028
> 5 years	2/46 (4.4%)	1/262 (0.4%)	–	–
Number of deliveries				
1 (primipara)	7/66 (10.6%)	2/396 (0.5%)	8.24 (1.38–49.37)	0.021
2 (multipara)	1/38 (2.6%)	1/228 (0.4%)	–	–
3 (multipara)	0/8	0/48	–	–

*aOR* adjusted odds ratio, *95% CI* 95% confidence interval.

## Discussion

This nationwide cohort study using the Korean NHI claims database found that gestational hypertension or preeclampsia was more common among kidney donors than in matched non-donors. However, the incidence of severe gestational hypertension or preeclampsia was similar between two groups. The time from donation to delivery within 5 years and primiparity were risk factors for preeclampsia. Other pregnant outcomes including low birth weight, stillbirth, and ectopic pregnancy did not differ significantly between the two groups. Although the rate of blood transfusion was significantly higher in kidney donors, there was no maternal death in kidney donors.

Young women donor candidates often ask if kidney donation will affect their future pregnancies. Two retrospective studies have reported an increased risk of gestational hypertension or preeclampsia in donors^13,14^. Thus, it is recommended that potential young donors should be counseled about the risk of gestational hypertension or preeclampsia^15^. Two previous studies have compared pregnancy outcomes before donation with outcomes after donation^14,18^. Furthermore, there is limited data on pregnancy outcomes in non-whited donors^19^. To the best of our knowledge, this is the first nationwide cohort study to evaluate the pregnancy outcomes in non-white donors.

The main strength of our study is that we evaluated all prescription data as well as diagnostic code in nationwide claims database. There is a lack of information regarding the clinical significance of gestational hypertension or preeclampsia in donors^9,16^. Previous studies have reported good maternal and fetal outcomes despite a higher incidence of gestational hypertension or preeclampsia, suggesting that the diseases that develops in donors is likely to be a mild form^13,14,18^. Confirming the incidence of gestational hypertension or preeclampsia based on the diagnostic code or questionnaires has the possibility of overestimation, especially in the antenatal care of living donors. Additionally, kidney donors may have increased proteinuria after donor nephrectomy, which may increase the likelihood of diagnosing preeclampsia. Garg et al. also supposed that the diagnosis of gestational hypertension or preeclampsia is more likely to be applied in kidney donors than in the general population under the same medical situation^13^. In this study, the incidence of gestational hypertension or preeclampsia was significantly higher in donors than in matched non-donors (8.9% vs. 1.8%), but the incidence of severe diseases requiring antihypertensive medications was comparable (0.9% vs. 2.7%). Further studies on pregnancy outcomes among donors are warranted to provide appropriate information to living donor candidates who plan for future pregnancy.

Hypertensive disorders of pregnancy including gestational hypertension or preeclampsia affect 2–8% in general population. Risk factors for disease development include maternal age, nulliparity, kidney disease, hypertension, obesity, and multiple pregnancy^12,25,26^. Given reduced kidney function and marginal increase of blood pressure after unilateral kidney donation, living donors are at higher risk for gestational hypertension or preeclampsia^27^. However, limited studies have analyzed risk factors in kidney donors and little evidence exist to guide the care of future pregnancies^9^. Kidney Disease: Improving Global Outcomes (KDIGO) clinical practice guideline suggests that the women with childbearing potential should be informed of the need to avoid becoming pregnant from the time of approval for donation to the time of recovery after donation^16^. However, there is no clear evidence to guide the time of pregnancies after kidney donation. The Ontario study found an increased risk of gestational hypertension or preeclampsia in donors > 32 years of age^13^. By contrast, recent study reported that first childbirth before 30 years of age is a risk factor for preeclampsia and eclampsia in donors^28^. In this study, multivariable analysis revealed that primipara and time from donation to delivery (< 5 years) were significantly associated with increased risk of preeclampsia. A plausible explanation for this finding is that initial significant reduction of glomerular filtration rate (GFR) after donation are gradually increased until five years attributable to compensatory hyperfiltration^29,30^. Our findings suggest that the importance of sufficient time from donation to recover and prenatal counseling for nulliparous potential donors.

Although this is a nationwide cohort study, it has several limitations. First, it was difficult to analyze pregnancy outcomes more precisely because laboratory data, such as kidney function and body mass index, were not available. Second, some donors may have a genetic predisposition to kidney disease, but this study could not investigate a family history. Third, in the analysis of risk factors, the number of cases of gestational hypertension and preeclampsia was small, limiting the statistical interpretation. Fourth, the operational definition of severe gestational hypertension with antihypertensive medication alone might overestimate the risk of severe disease. Detailed prescription data were not able to utilize due to the inherent limitations of nationwide claims database.

In summary, our findings indicate that living kidney donors may be associated with an increased risk of gestational hypertension or preeclampsia compared to matched healthy non-donors. However, incidence of severe gestational hypertension or preeclampsia that required antihypertensive medication was confirmed in less than 3% of kidney donors, and no maternal death was reported. Additionally, this study shows that the probabilities of the most serious maternal and fetal outcomes remain low and do not increase significantly after kidney donation. Therefore, it is necessary to sufficiently inform and discuss the risks associated with the above before donating a kidney to a living kidney donor of childbearing age.

## Data Availability

The datasets used and/or analysed during the current study available from the corresponding author on reasonable request.
